# Behind the Smoke: A Bladder Cancer Case Report Through the Eyes of Primary Care

**DOI:** 10.7759/cureus.82435

**Published:** 2025-04-17

**Authors:** Inês Bento, José Freitas, Bárbara Campos, Telmo Fonseca, Cristiana Leite

**Affiliations:** 1 Family Medicine, Unidade Local de Saúde de Gaia/Espinho, Vila Nova de Gaia, PRT

**Keywords:** bladder carcinoma, incidentaloma, occupational exposure, primary medical care, screening, smoking

## Abstract

Bladder carcinoma represents a common neoplasm in men, typically presenting with hematuria or as an incidental finding. Smoking and occupational exposure are its main risk factors. Currently, no screening method is available for its detection.

We present the case of a 69-year-old retired man (former car mechanic), with a history of hypertension, dyslipidemia, and type 2 diabetes mellitus, who attended a routine appointment at his primary healthcare center, with no complaints. In addition to standard tests, an abdominal ultrasound was requested to screen for abdominal aneurysm, in accordance with established guidelines, as the patient was a heavy smoker. Three weeks later, he returned with his test results, identifying two suspicious polypoid formations in the bladder. He was referred to a urology consultation, where he underwent a transurethral resection of the bladder tumor. Posterior examination identified two carcinomas, requiring chemotherapy for one year.

This case describes an asymptomatic patient with risk factors for bladder cancer who was diagnosed incidentally, emphasizing the need for their careful management, a crucial role played by family physicians.

## Introduction

Bladder cancer is the 10th most common cancer worldwide and the sixth most prevalent in men. Non-invasive urothelial carcinoma accounts for the majority of cases, presenting with painless hematuria, with lower urinary tract symptoms, or as an incidental finding [1]. Globally, in 2020, there were approximately 213,000 deaths attributed to bladder cancer, with a higher incidence in Southern European countries [2].

Most bladder cancers are associated with external risk factors, with smoking (linked to about half of bladder cancer cases) and occupational exposure being the primary ones [1,3]. In a cohort study involving 422,010 participants, smoking was associated with a twofold to threefold increased risk of bladder cancer (hazard ratios of 2.32 for men and 2.75 for women) [4]. An estimated 5.7% of new bladder cancer cases are attributed to occupational exposure to carcinogens, particularly in professions such as chimney sweeps, hairdressers, automobile drivers, and firefighters [1]. Moreover, recent studies have demonstrated a correlation between diesel exhaust exposure and bladder cancer, with cumulative exposure to respirable elemental carbon being linked to an elevated risk of urothelial bladder cancer (odds ratio of 1.61) [5].

While the prognosis for non-muscle-invasive bladder cancer is relatively favorable, particularly in lower-risk groups, long-term survival outcomes for muscle-invasive bladder cancer are significantly poorer. In fact, the long-term outlook for metastatic urothelial carcinoma remains bleak, with a five-year survival rate of approximately 5% [6]. The strong correlation between disease stage and prognosis in bladder cancer supports the idea that early detection and prompt treatment, preferably before muscle invasion, could be an effective approach to lowering its high mortality rate [7]. Therefore, timely detection of bladder cancer might enable less invasive treatment and improve overall outcomes in terms of morbidity and mortality [8].

Several scientific societies recommend smoking cessation and referral for cystoscopy in cases of suspicious symptoms as the best approach to preventing bladder cancer. However, for asymptomatic individuals, there is insufficient evidence to support screening [9]. This case report aims to raise awareness about recognizing and managing asymptomatic patients with high-risk factors, a key responsibility of family physicians.

## Case presentation

We present the case of a 69-year-old married man (retired former car mechanic). His medical history included hypertension (HTN), dyslipidemia, and type 2 diabetes mellitus (DM), managed with valsartan/hydrochlorothiazide 160/12.5 mg, lercanidipine 10 mg, simvastatin 40 mg, fenofibrate 145 mg, and metformin 1000 mg. He had a smoking history of 20 cigarettes per day since the age of 18 (51 pack-years), with previous unsuccessful attempts at smoking cessation. His alcohol consumption was 65 g/day, and he was under follow-up at the local Alcoholism Treatment Center. Additionally, he drank eight cups of coffee daily. He had no known drug or food allergies. Family history included a brother who died of liver disease, though details were unclear.

His family unit consisted of his wife and two children, classified as a nuclear family in stage VIII of the Duvall cycle and class IV on the Graffar scale (lower middle class) [10]. The family was highly functional, scoring 9 points on the Smilkstein Family Adaptation, Partnership, Growth, Affection, and Resolve (APGAR) [11].

On October 31, 2024, the patient attended a routine follow-up appointment at his primary healthcare (PHC) center for DM and HTN management. He was asymptomatic. His medical history revealed several risk factors and previous occupational exposure to exhaust gases, which had been considered in previous consultations for opportunistic screenings, including a chest X-ray and spirometry (for lung disease detection), fecal occult blood test (for colorectal cancer detection), and the Fracture Risk Assessment Tool (FRAX) questionnaire (for osteoporosis screening). In this consultation, an abdominal ultrasound was requested to screen for an aortic aneurysm (given smoking as a risk factor), along with blood and urine tests for DM and HTN monitoring.

**Figure 1:**
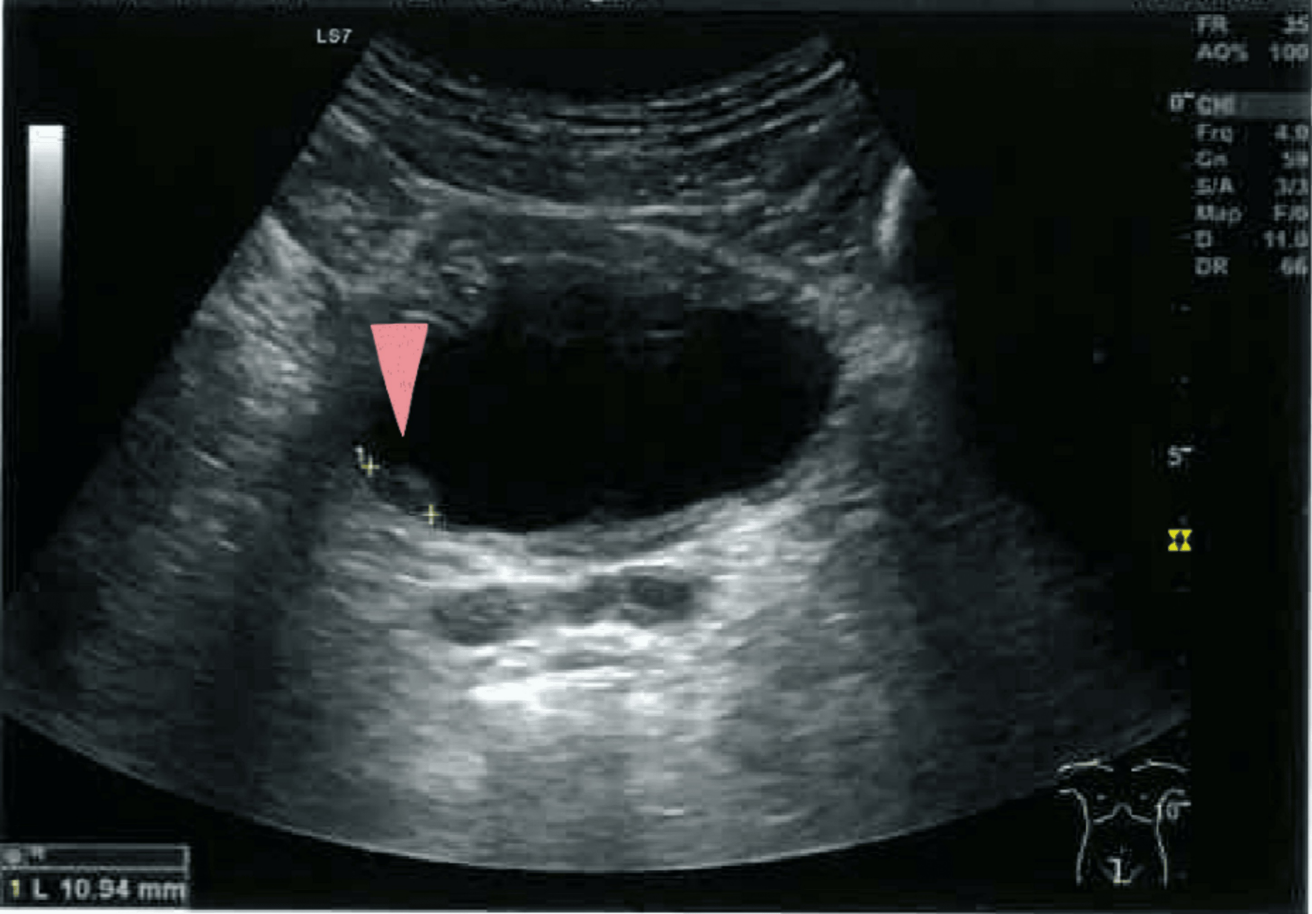
Polypoid formation in the bladder wall, measuring 10.94 mm

**Figure 2:**
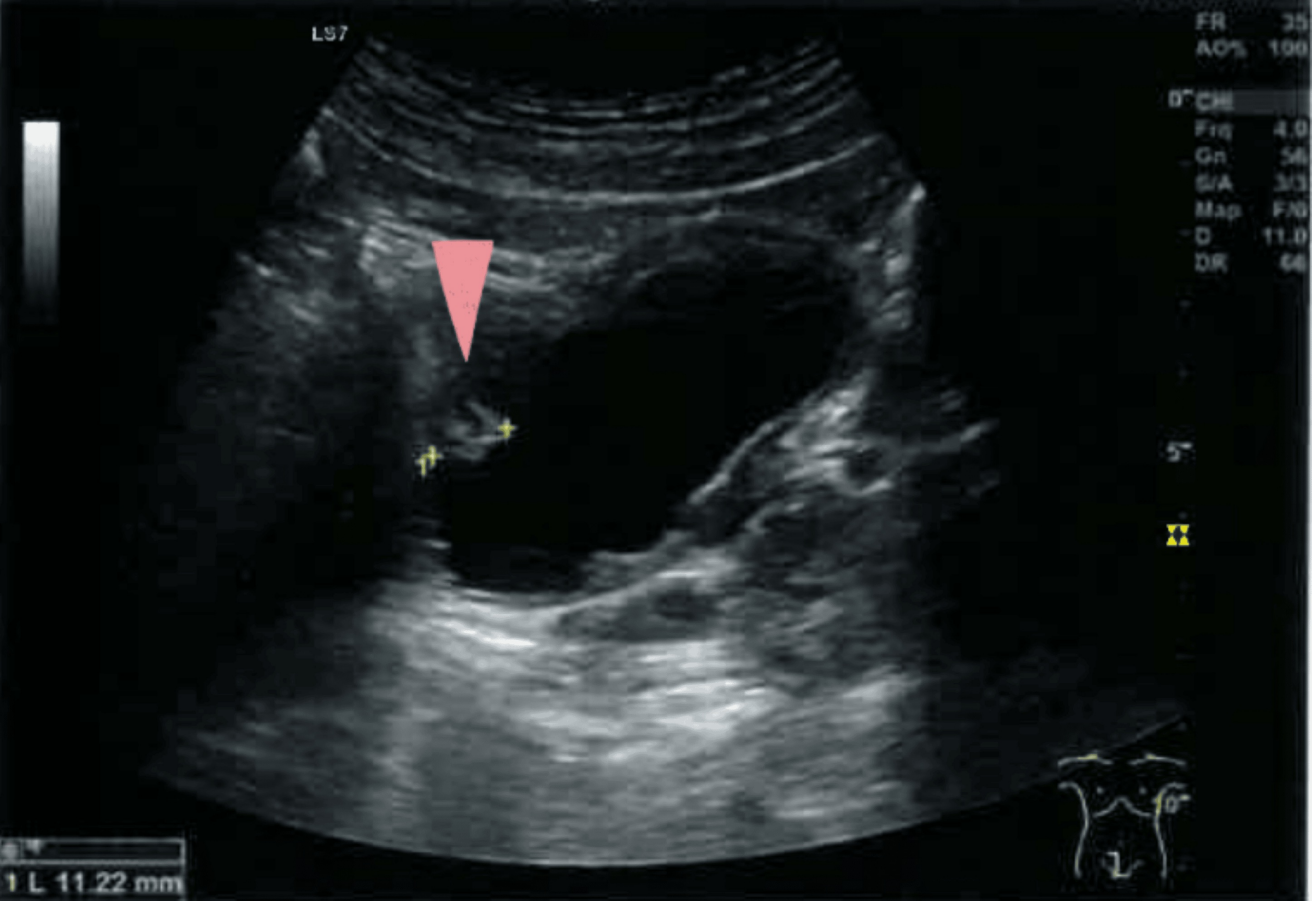
Second polypoid formation in the bladder wall, measuring 11.22 mm

Three weeks later, he returned to his PHC for test results evaluation. The laboratory results were normal. The urine analysis showed 2-5 red blood cells per field, and the abdominal ultrasound identified two polypoid formations in the bladder wall, superior and lower right side, measuring 11 and 10 mm in diameter (Figure 1 and Figure 2). Due to the suspicious nature of the formations, the patient was referred to a urology consultation for further investigation.

Three months later, in January 2025, he was reassessed in a scheduled PHC visit, reporting that he had undergone complete transurethral bladder resection. Posterior investigation by the urology consultation revealed two superficial tumors on the posterior bladder wall, consistent with urothelial carcinoma. Following a multidisciplinary meeting, chemotherapy with mitomycin C instillations was prescribed for one year, with a urology follow-up scheduled in three months. The patient remained asymptomatic throughout the process.

## Discussion

This case describes a patient with significant risk factors for bladder cancer (smoking and occupational exposure to exhaust gases) who never exhibited urinary symptoms. During medical history review, screenings for various neoplasms were considered; however, bladder cancer was not initially included in the family physician's assessments. The absence of screening recommendations, even in high-risk adults, along with the lack of symptoms, explains this omission.

According to the United States Preventive Services Task Force (USPSTF), the quality of evidence on the benefits, risks, and diagnostic capability of screening tests (urinalysis for microscopic hematuria, cytology, or urinary biomarkers) is insufficient, preventing their recommendation for bladder cancer screening in asymptomatic adults [9]. However, the two main risk factors can be identified during routine consultations with family physicians, who can engage in secondary prevention and targeted follow-up. Additionally, given the potential risk of underlying urological conditions, the presence of microscopic hematuria in a patient with risk factors may warrant further evaluation, despite the lack of clear guidelines.

According to the World Organization of Family Doctors (WONCA), a core competency of General and Family Medicine is healthcare management, which includes "the efficient use of healthcare resources, in coordination with other specialties". Additionally, another key competency is managing conditions that may arise early or in an undifferentiated manner [12]. As primary healthcare providers, family physicians should focus on identifying risk factors and promoting appropriate secondary prevention, including urinalysis for microscopic hematuria detection. This case highlights the crucial role of family physicians in patient prognosis, both in gathering a detailed medical history and in coordinating with specialists for timely intervention.

## Conclusions

This case underscores the critical role of family physicians in identifying risk factors for bladder cancer, particularly in patients with a history of smoking and occupational exposure. While screening for bladder cancer in asymptomatic adults is not currently recommended due to insufficient evidence, family physicians can still play an essential role in secondary prevention. By reviewing a patient's medical history, recognizing key risk factors, and promoting targeted follow-up, family physicians contribute significantly to early detection and improved patient outcomes. The coordination of resources and proactive management highlights the importance of family physicians in optimizing patient care.
